# Factors Associated With Viral Suppression and Drug Resistance in Children and Adolescents Living With HIV in Care and Treatment Programs in Southern Tanzania

**DOI:** 10.1093/jpids/piad040

**Published:** 2023-06-03

**Authors:** Samoel A Khamadi, Emmanuel Bahemana, Nicole Dear, Caroline Mavere, Fredy George, Razack Kapene, Grace Papianus, Walidah Willoughby, Jillian Chambers, Kavitha Ganesan, Iman Mwakabanje, Jason M Bacha, Priyanka Desai, Shaban Almas, Peter D Coakley, Vanessa Wolfman, Elizabeth H Lee, Patrick W Hickey, Jeffrey Livezey, Patricia A Agaba

**Affiliations:** Walter Reed Program/HJF Medical Research International, Mbeya, Tanzania; Walter Reed Program/HJF Medical Research International, Mbeya, Tanzania; U.S. Military HIV Research Program, Walter Reed Army Institute of Research, Silver Spring, Maryland, USA; Henry M. Jackson Foundation for the Advancement of Military Medicine, Bethesda, Maryland, USA; Walter Reed Program/HJF Medical Research International, Mbeya, Tanzania; Walter Reed Program/HJF Medical Research International, Mbeya, Tanzania; Walter Reed Program/HJF Medical Research International, Mbeya, Tanzania; Walter Reed Program/HJF Medical Research International, Mbeya, Tanzania; U.S. Military HIV Research Program, Walter Reed Army Institute of Research, Silver Spring, Maryland, USA; Henry M. Jackson Foundation for the Advancement of Military Medicine, Bethesda, Maryland, USA; U.S. Military HIV Research Program, Walter Reed Army Institute of Research, Silver Spring, Maryland, USA; Henry M. Jackson Foundation for the Advancement of Military Medicine, Bethesda, Maryland, USA; U.S. Military HIV Research Program, Walter Reed Army Institute of Research, Silver Spring, Maryland, USA; Henry M. Jackson Foundation for the Advancement of Military Medicine, Bethesda, Maryland, USA; Walter Reed Program/HJF Medical Research International, Mbeya, Tanzania; Baylor College of Medicine, Department of Pediatrics, Houston, Texas, USA; Baylor College of Medicine International Pediatric AIDS Initiative (BIPAI) at Texas Children’s Hospital, Baylor College of Medicine, Houston, Texas, USA; Baylor College of Medicine Children’s Foundation—Tanzania, Mbeya, Tanzania; U.S. Military HIV Research Program, Walter Reed Army Institute of Research, Silver Spring, Maryland, USA; Henry M. Jackson Foundation for the Advancement of Military Medicine, Bethesda, Maryland, USA; Walter Reed Program/HJF Medical Research International, Mbeya, Tanzania; U.S. Military HIV Research Program, Walter Reed Army Institute of Research, Silver Spring, Maryland, USA; Henry M. Jackson Foundation for the Advancement of Military Medicine, Bethesda, Maryland, USA; U.S. Military HIV Research Program, Walter Reed Army Institute of Research, Silver Spring, Maryland, USA; Henry M. Jackson Foundation for the Advancement of Military Medicine, Bethesda, Maryland, USA; The Uniformed Services University of the Health Sciences, Bethesda, Maryland, USA; The Uniformed Services University of the Health Sciences, Bethesda, Maryland, USA; The Uniformed Services University of the Health Sciences, Bethesda, Maryland, USA; U.S. Military HIV Research Program, Walter Reed Army Institute of Research, Silver Spring, Maryland, USA; Henry M. Jackson Foundation for the Advancement of Military Medicine, Bethesda, Maryland, USA

**Keywords:** children and adolescents, drug resistance, integrase inhibitors, viral suppression

## Abstract

**Background:**

Achieving viral suppression (VS) for persons living with HIV is key to reaching epidemic control. We assessed the prevalence of VS and the frequency of HIV drug resistance mutations (HIVDRM) among children and adolescents living with HIV (CALHIV) in the Southern Highland zone of Tanzania.

**Methods:**

From 2019 to 2021, we enrolled CALHIV aged 1–19 years on ART for >6 months in a cross-sectional study. Participants had viral load (VL) testing; those with VL ≥ 1000 copies/mL underwent HIVDRM testing. VS (<1000 copies/mL) prevalence estimates were calculated and robust Poisson regression was used to estimate prevalence ratios (PRs) and 95% confidence intervals (CIs) for associations with potential predictors of VS.

**Results:**

Of 707 participants, 595 had VS (PR: 0.84, 95% CI: 0.81–0.87). Use of an integrase strand transfer inhibitor-containing regimen (aPR 1.15, 95% CI: 0.99–1.34), age 5–9 years (aPR 1.16, 95% CI: 1.07–1.26), and seeking care at a referral center (aPR 1.12, 95% CI: 1.04–1.21) were associated with VS. Factors inversely associated with VS included having one (aPR 0.82, 95% CI: 0.72–0.92) or two or more (aPR 0.79, 95% CI: 0.66–0.94) referrals for adherence counselling, and self-reporting missing one to two (aPR 0.88, 95% CI: 0.78–0.99) or three or more (aPR 0.77, 95% CI: 0.63–0.92) doses of ART in the past month. Of 74 participants with PRRT and INT sequencing done, 60 (81.1%) had HIVDRMs at the following frequencies: 71.6%, 67.6%, 1.4%, and 4.1% for major NNRTI, NRTI, PI, and INSTI respectively.

**Conclusions:**

Higher rates of VS were observed in this cohort, and HIVDRMs were common in those without VS. This evidence supports ART optimization using dolutegravir-based regimens. However, better strategies to improve adherence are needed.

## BACKGROUND

In 2020, the Joint United Nations Program on HIV/AIDS (UNAIDS) estimated that there were 2.78 million children and adolescents living with HIV (CALHIV) globally, and they accounted for 10% of new infections and 15% of AIDS-related deaths [1]. However, pediatric treatment coverage has lagged behind coverage for adults. While 74% of those aged 15 years and older were on antiretroviral therapy (ART) in 2021, only 52% of children were on treatment [2]. Early initiation of ART reduces morbidity and mortality and improves life expectancy and quality of life. Low coverage of this critical intervention among CALHIV limits their ability to achieve a long and healthy life [3].

The goal of ART is durable viral suppression (VS), with subsequent restoration of the immune system, and reduction in mortality and HIV transmission [4–6]. In 2014, UNAIDS set ambitious targets to achieve epidemic control in 2020 through the 90-90-90 initiative [7]. Although these targets were subsequently increased to 95-95-95 by 2030 that is diagnose 95% of all HIV-positive individuals, retain 95% of those diagnosed on treatment and achieve viral suppression for 95% of those treated [5], many countries continue to struggle with attaining the third ‘95 target among CALHIV. Following World Health Organization (WHO) recommendations, viral load (VL) testing became the standard of care for monitoring treatment efficacy in 2013, with national programs adjusting their HIV treatment guidelines to facilitate earlier detection of treatment failure [8–11]; the government of Tanzania adopted routine VL monitoring as the standard of care for all clients on ART in 2015 [12].

Failure to attain the UNAIDS third “95” target among CALHIV continues to pose a challenge to epidemic control, as studies have reported lower VS rates in this sub-population compared to adults [13, 14], with various factors being documented as responsible for poor VS. Prior Tanzanian studies have documented VS rates for CALHIV ranging between 60 and 68% [15, 16]. These studies were conducted in the period when non-nucleoside reverse transcriptase inhibitor (NNRTI)-based regimens were the preferred first-line for treatment [17]. Current national guidelines recommend dolutegravir (DTG), in combination with tenofovir (TDF) and lamivudine (3TC), a single fixed-dose combination tablet (TDF/3TC/DTG) as the preferred first-line regimen for CALHIV weighing 30 kg and above. For younger children, the preferred first-line regimens are a combination of abacavir (ABC), 3TC and lopinavir boosted with ritonavir (LPV/r) for CALHIV weighing <20 kg and ABC, 3TC, and DTG for those weighing between 20 and 29.9 kg. For second-line options, the preferred regimen for CALHIV weighing ≥20 kg and above is zidovudine (AZT), 3TC and ritonavir-boosted atazanavir (ATV/r), with the recommendation to maintain the protease-inhibitor (PI) base while titrating the nucleoside reverse transcriptase inhibitor (NRTI) component for CALHIV <20 kg [18, 19]. As DTG-based regimens for CALHIV have been rolled out in Tanzania since 2019, updated data on the prevalence of, and factors associated with, VS and HIVDRM in CALHIV are needed.

Our primary objective was to estimate the prevalence of VS among CALHIV in the Southern Highlands of Tanzania. Additionally, the study sought to identify factors associated with VS and describe HIVDRMs occurring among participants.

## METHODS

### Study Population

Random sampling was conducted on lists of eligible participants on ART between 2019 and 2021 at 15 treatment clinics (Supplementary Figure) supported by the United States Military HIV Research Program (MHRP) and The U.S. President’s Emergency Plan for AIDS Relief (PEPFAR) in Tanzania. The random sample was weighted by clinic and stratified by duration of ART and age. Randomization was done in STATA using listings of current clients from each clinic and is representative of the general population of CALHIV on ART. Eligible CALHIV had to be 1–19 years of age; have been on first- or second-line ART for at least 6 months; and had attended at least one follow-up visit in the last 6 months. Participants aged 13–17 years were required to have been informed of their HIV status in order to provide assent for participation. Participants identified from the randomization lists were contacted by study teams and invited to enroll in this cross-sectional study.

### Data Collection

Participants underwent a blood draw for HIV VL unless test results for routine clinical care within 1 month of study enrollment were available. We also included viral loads that were outside the original 1 month window for participants without a recorded viral load value within the month prior to enrollment. During the study visit, study staff administered sociodemographic questionnaires to participants aged ≥18 years, to caregivers if the participant was younger than 13 years, and to both caregivers and participants if the participant was between 13 and 17 years. Information on the clinical history and ART records from written files, electronic clinical and pharmacy systems, and ART registers for each participant were abstracted by the study team within 3 weeks of the participant’s visit. Data from medical record abstraction captured information and events prior to enrollment into the study. Referral history captured documented referrals for adherence counselling and other relevant support services documented in the charts. However, source documentation did not include information on whether participants received the services for which they were referred. Data were transcribed onto case report forms and entered into the Clinplus platform (Anju Software, Tempe, AZ).

### Ethical Assurance

The study was approved by institutional review boards of the Walter Reed Army Institute of Research, Baylor College of Medicine, Mbeya Medical Research and Ethics Committee, and the Tanzania National Institute for Medical Research. All participants provided informed consent and assent, as applicable.

### Viral Load Monitoring

HIV VL was measured via nucleic acid amplification methods on the Abbott m2000sp/rt RealTime HIV-1 assay (Abbott Laboratories, Chicago, IL) System with a reportable range of –10 000 000 copies/mL.

### HIV Genotyping and Subtyping

Plasma samples from all participants with VL ≥ 1000 copies/mL underwent sequencing of the Pol region using a laboratory-validated modification to the ViroSeq HIV-1 Genotyping System v2.0 (Abbott Molecular, Chicago, IL). Sequences were evaluated for major mutations conferring resistance to nucleoside reverse transcriptase inhibitors (NRTIs), NNRTIs, protease inhibitors (PIs), and integrase strand transfer inhibitors (INSTI) using the SmartGene Integrated Database Network System (SmartGene, Zug, Switzerland) to access mutation lists from the Stanford HIV Drug Resistance Database Version 8.8.0 (Stanford University, Stanford, CA).

Extraction of viral ribonucleic acid (RNA) from the samples was performed using Purelink™ Viral deoxyribonucleic acid (DNA)/RNA Minikit, and HIV genotyping was performed using ThermoFisher HIV-1 Genotyping kit with Integrase—for PR, RT, and INT genes. GeneAmp® 9700 polymerase chain reaction (PCR) system thermocyclers were used for PCR procedures, and final sequence detection was performed using ABI 3500xL Genetic Analyzer.

Sequence raw data were analyzed using online RECall software (version 2.32) for reviewing base-calling, sequence editing, and generation of consensus sequences. For sequence quality control (QC), MEGA-X software (Version 10.2) was used for assessing genetic distance among the samples by pairwise alignment. HIV subtyping QC was performed using REGA, National Center for Biotechnology Information (NCBI), Stanford and Jumping HIV subtyping tools, and the generation of final HIV drug resistance reports was done by using the Integrated Database Network System (IDNS) Smart gene software.

HIV-1 subtype was inferred from the consensus evolutionary tree from SmartGene IDNS, which utilizes the neighbor-joining method in MEGA4 software. Evolutionary distances were computed using the maximum composite likelihood method in units of the number of base substitutions per site. The tree was then generated by the neighbor-joining method from a nucleotide alignment. All testings for VL and HIVDRMs were performed according to the manufacturer’s instructions at the Mbeya Zonal Referral Hospital Laboratory in Mbeya region, Tanzania.

### Statistical Analyses

VS was defined as VL < 1000 copies/mL [8]. The prevalence of VS was estimated using the Wilson score method and reported with 95% confidence intervals (CIs) in the overall sample, and by sex, age group, duration on ART, first versus second-line ART, and ART class. Bivariate analyses were conducted using Pearson’s chi-squared tests. Generalized linear models with a Poisson distribution and robust standard errors were used to estimate unadjusted and adjusted prevalence ratios (aPRs) and 95% CIs for associations between sociodemographic and clinical factors and VS. Individual-level predictors determined to be significant (*α* < 0.05) in the unadjusted models and those identified based on *a priori* and clinical knowledge of the study setting were included in the fully adjusted model. Predictors identified a priori included sex, age, and duration of ART and ART class. We tested for multicollinearity using the variable inflation factor; no variables were found to be collinear. Additional predictors identified in bivariate analyses were included in the adjusted models. Analyses were restricted to complete cases after creating separate categories for unknown data from participant medical records (WHO disease staging, nutritional status, and history of TB treatment). For participants with a VL ≥ 1000 copies/mL, the prevalence of specific HIVDRMs and categories of HIVDRMs were calculated by dividing the number of participants with one or more mutations by the total number of participants genotyped.

Analyses were performed in Statistical Analysis System (SAS) version 9.4 (SAS Institute, Cary, NC) and Stata version 16.1 (StataCorp, College Station, TX) software.

## RESULTS

### Participant Characteristics

Randomization identified 785 CALHIV as potential participants for the study and was screened for eligibility, of which 774 were fully eligible for enrollment. Of those 774 and 747 were successfully contacted by the study team, agreed to participate, and were enrolled. However, only 707 (94.6%) had complete case data and were included in further analyses. The median age was 12 (interquartile range-IQR: 9–16) years and 382 (54.0%) were female (Table 1). Twenty-eight (4.0%) had been on ART for less than a year, 63 (8.9%) for 1–2 years, and 616 (87.1%) for more than 2 years. The median duration of the current ART regimen was 1.1 (IQR: 0.5–1.7) years.

**Table 1.** Characteristics of Children and Adolescents Attending MHRP/PEPFAR-Supported Antiretroviral Treatment Programs in Tanzania by Viral Suppression Status

	Viral Load, <1000 c/mL (*n* = 595)	Viral Load, ≥1000 c/mL (*n* = 112)	Total (*N* = 707)	*P*-Value
**Sex**				.600
** Male**	271 (45.5%)	54 (48.2%)	325 (46.0%)	
** Female**	324 (54.5%)	58 (51.8%)	382 (54.0%)	
**Age (years)**				**.012**
** 1–4**	27 (4.5%)	12 (10.7%)	39 (5.5%)	
** 5–9**	171 (28.7%)	20 (17.9%)	191 (27.0%)	
** 10–14**	204 (34.3%)	41 (36.6%)	245 (34.7%)	
** 15–19**	193 (32.4%)	39 (34.8%)	232 (32.8%)	
**Duration on ART**				.690
** 6–12 months**	25 (4.2%)	3 (2.7%)	28 (4.0%)	
** 13–24 months**	54 (9.1%)	9 (8.0%)	63 (8.9%)	
** >24 months**	516 (86.7%)	100 (89.3%)	616 (87.1%)	
**Current NRTI backbone** ^1^				.620
** ABC/3TC backbone**	244 (41.0%)	46 (41.1%)	290 (41.0%)	
** AZT/3TC backbone**	43 (7.2%)	11 (9.8%)	54 (7.6%)	
** TDF/3TC backbone**	308 (51.8%)	55 (49.1%)	363 (51.3%)	
**Current ART class** ^2^				**.002**
** NNRTI-based**	43 (7.2%)	13 (11.6%)	56 (7.9%)	
** PI-based**	114 (19.2%)	35 (31.3%)	149 (21.1%)	
** INSTI-based**	438 (73.6%)	64 (57.1%)	502 (71.0%)	
**ART Line** ^3^				.810
** 1st line**	342 (57.5%)	63 (56.3%)	405 (57.3%)	
** 2nd line**	253 (42.5%)	49 (43.8%)	302 (42.7%)	
**ART regimen change in past 6 months**				.460
** No**	445 (74.8%)	80 (71.4%)	525 (74.3%)	
** Yes**	150 (25.2%)	32 (28.6%)	182 (25.7%)	
**Adherence counseling referrals** ^4^				**<.001**
** Never referred**	486 (81.7%)	69 (61.6%)	555 (78.5%)	
** One referral**	74 (12.4%)	29 (25.9%)	103 (14.6%)	
** 2 or more referrals**	35 (5.9%)	14 (12.5%)	49 (6.9%)	
**Most recently documented WHO clinical stage** ^5^				.490
** I**	80 (13.4%)	11 (9.8%)	91 (12.9%)	
** II**	142 (23.9%)	33 (29.5%)	175 (24.8%)	
** III**	224 (37.6%)	45 (40.2%)	269 (38.0%)	
** IV**	81 (13.6%)	14 (12.5%)	95 (13.4%)	
** Unknown**	68 (11.4%)	9 (8.0%)	77 (10.9%)	
**Most recently documented nutritional status** ^6^				.390
** No malnutrition**	527 (88.6%)	104 (92.9%)	631 (89.3%)	
** Moderate/severe malnutrition**	6 (1.0%)	1 (0.9%)	7 (1.0%)	
** Unknown**	62 (10.4%)	7 (6.3%)	69 (9.8%)	
**History of TB treatment** ^7^				.430
** No**	526 (88.4%)	95 (84.8%)	621 (87.8%)	
** Yes**	47 (7.9%)	13 (11.6%)	60 (8.5%)	
** Unknown**	22 (3.7%)	4 (3.6%)	26 (3.7%)	
**Ever experienced side effects** ^8^				.890
** No**	434 (72.9%)	81 (72.3%)	515 (72.8%)	
** Yes**	161 (27.1%)	31 (27.7%)	192 (27.2%)	
**Self-reported frequency of taking medication** ^9^				**.032**
** Once per day**	417 (70.1%)	67 (59.8%)	484 (68.5%)	
** Twice or more per day**	178 (29.9%)	45 (40.2%)	223 (31.5%)	
**Self-reported missed doses of ART in past month** ^10^				**<.001**
** None**	495 (83.2%)	75 (67.0%)	570 (80.6%)	
** 1–2**	66 (11.1%)	20 (17.9%)	86 (12.2%)	
** 3 or more**	34 (5.7%)	17 (15.2%)	51 (7.2%)	
**Self-reported having taken ART continuously in past 6 months** ^11^				.068
** No**	52 (8.7%)	16 (14.3%)	68 (9.6%)	
** Yes**	543 (91.3%)	96 (85.7%)	639 (90.4%)	
**Level of care delivery**				**.008**
** Primary health facility**	180 (30.3%)	40 (35.7%)	220 (31.1%)	
** District health facility**	136 (22.9%)	35 (31.3%)	171 (24.2%)	
** Regional health facility**	70 (11.8%)	16 (14.3%)	86 (12.2%)	
** Referral health center**	209 (35.1%)	21 (18.8%)	230 (32.5%)	
**Clinic support group participation among those 13+ years old (*n* = 342)** ^12^				**.007**
** Attends**	47 (78.3%)	169 (59.9%)	216 (63.2%)	
** Does not attend**	13 (21.7%)	113 (40.1%)	126 (36.8%)	
**Community support group participation among those 13+ years old (*n* = 342)** ^13^				.420
** Attends**	4 6.7%)	12 4.3%)	16 4.7%)	
** Does not attend**	56 (93.3%)	270 (95.7%)	326 (95.3%)	

Data are presented as n (column %). Bold values indicate significance at *P* < .05. Abbreviations: ABC, abacavir; AZT, azidothymidine (zidovudine); 3TC, lamivudine; TDF, tenofovir. Pearson’s chi-squared tests were used to compare participants with viral load <1000 copies/mL and those with viral load ≥1000 copies/mL. *P*-values were not corrected for multiple-hypothesis testing. Facilities participating in this study include Town Clinic Health Center, Mpanda District Hospital, Ipinda Health Center, Tunduma Health Center, Kyela District Hospital, Baylor Center of Excellence MZRH, Mbeya Regional Referral Hospital, Mazwi Health Center, Laela Health Center, Dr. Atman Hospital, Sumbawanga Hospital, Mjimwema Health Center, Matarawe Dispensary, Mbinga District Hospital, and Songea Regional Referral Hospital.

^1^Current NRTI backbone: ART regimen was obtained by medical and pharmacy record abstraction and grouped by common regimen/regimen backbone.

^2^ART class: ART class was derived from ART regimen data obtained by medical and pharmacy record abstraction.

^3^ART line: 1st vs 2nd line ART class was derived from ART regimen variable.

^4^Number of referrals for adherence counseling: adherence counseling referral data were obtained by medical record abstraction.

^5^Most recently documented WHO clinical stage: WHO staging was obtained by medical record abstraction at the most recent clinical care visit.

^6^Most recently documented nutritional status: nutritional status was obtained by medical record abstraction at the most recent clinical care visit and categorized according to the WHO classification scheme for malnutrition.

^7^History of tuberculosis (TB) treatment: history of having ever received TB treatment was obtained by medical record abstraction.

^8^Ever experienced side effects: participants 18–19 years, caregivers if the participant was younger than 13 years, and both caregivers and participants if the participant was between 13 and 17 years old were asked if the participant had ever experienced side effects as a result of ART treatment; for participants 13–17 years, the caregiver response was taken as the primary response; however, if the caregiver response was missing, the participant response was utilized.

^9^Self-reported frequency of taking medication: participants 18–19 years, caregivers if the participant was younger than 13 years, and both caregivers and participants, if the participant was between 13 and 17 years old were asked how frequently the participant takes medication for HIV; for participants 13–17 years, the caregiver response was taken as the primary response; however, if the caregiver response was missing, the participant response was utilized.

^10^Self-reported doses of ART missed in the past month: adolescents aged 18–19 years, caregivers if the participant was younger than 13 years, and both caregivers and participants if the participant was between 13 and 17 years were asked how many self-reported doses of ART medication the participant had missed over the last month; responses were categorized into none, 1–2 or 3 or more missed ART doses in the past month; for participants 13–17 years, the caregiver response was taken as the primary response; however, if the caregiver response was missing, the participant response was utilized.

^11^Self–reported having taken ART continuously in the past 6 months: adolescents aged 18–19 years, caregivers if the participant was younger than 13 years, and both caregivers and participants, if the participant was between 13 and 17 years were asked if the participant had taken ART continuously in the past 6 months, with no treatment interruptions greater than 1 week; for participants 13–17 years, the caregiver response was taken as the primary response; however, if the caregiver response was missing, the participant response was utilized.

^12^Clinic support group participation: children and adolescents 13–19 years were asked if they participate in a support group at the clinic; children under age 13 were considered too young to participate as disclosure in this age group was not required for enrollment.

^13^Community support group participation: children and adolescents 13–19 years were asked if they participate in an HIV treatment support group in their community; children under age 13 were considered too young to participate as disclosure in this age group was not required for enrollment.

At enrollment, a total of 290 (41.0%) participants were on an ART regimen with an abacavir ABC/3TC backbone, 54 (7.6%) were on an AZT/3TC backbone, and 363 (51.3%) were on a TDF/3TC backbone regimen, including 339 (93.4%) who were on TDF/3TC/DTG. There were 405 (57.3%) on first-line and 302 (42.7%) on second-line therapy.

A total of 103 (14.6%) participants had documented a history of one referral for adherence counselling, while 49 (6.9%) had been referred two or more times for adherence counselling. Referrals for other services, not including adherence counseling, were documented for 235 (33.2%) participants. At the time of study enrollment, 631 (89.3%) were well nourished and 570 (80.6%) self-reported no missed doses of ART in the month preceding enrollment. The distribution of ART use across the age bands revealed significant differences in the current ART class and current NRTI backbone by age (Table 2). ART regimens prescribed differed by weight band, with most children under 20 kg on ABC/3TC/LPV/r, most children between 20 and 20.9 kg on ABC/3TC/DTG and most children and adolescents 30 kg and above on TDF/3TC/DTG (Supplementary Table).

**Table 2.** Select Characteristics of Children and Adolescents Attending MHRP/PEPFAR-Supported Antiretroviral Treatment Programs in Tanzania by Age Group

	1–4 Years (*n* = 39)	5–9 Years (*n* = 191)	10–14 Years (*n* = 245)	15–19 years (*n* = 232)	Total (*N* = 707)	*P*-Value
Current NRTI backbone						**<.001**
ABC/3TC backbone	35 (89.7%)	157 (82.2%)	79 (32.2%)	19 (8.2%)	290 (41.0%)	
AZT/3TC backbone	4 (10.3%)	26 (13.6%)	19 (7.8%)	5 (2.2%)	54 (7.6%)	
TDF/3TC backbone	0 (0.0%)	8 (4.2%)	147 (60.0%)	208 (89.7%)	363 (51.3%)	
Current ART class						**<.001**
NNRTI-based	3 (7.7%)	31 (16.2%)	16 (6.5%)	6 (2.6%)	56 (7.9%)	
PI-based	33 (84.6%)	63 (33.0%)	20 (8.2%)	33 (14.2%)	149 (21.1%)	
INSTI-based	3 (7.7%)	97 (50.8%)	209 (85.3%)	193 (83.2%)	502 (71.0%)	
ART Line						**<.001**
1st line	13 (33.3%)	39 (20.4%)	157 (64.1%)	196 (84.5%)	405 (57.3%)	
2nd line	26 (66.7%)	152 (79.6%)	88 (35.9%)	36 (15.5%)	302 (42.7%)	
Self-reported missed doses of ART in past month						.058
None	24 (61.5%)	158 (82.7%)	205 (83.7%)	183 (78.9%)	570 (80.6%)	
1–2	9 (23.1%)	19 (9.9%)	26 (10.6%)	32 (13.8%)	86 (12.2%)	
3 or more	6 (15.4%)	14 (7.3%)	14 (5.7%)	17 (7.3%)	51 (7.2%)	
Self-reported having taken ART continuously in past 6 months						.880
No	3 (7.7%)	21 (11.0%)	23 (9.4%)	21 (9.1%)	68 (9.6%)	
Yes	36 (92.3%)	170 (89.0%)	222 (90.6%)	211 (90.9%)	639 (90.4%)	
Viral load						**.012**
≥1000 copies/mL	12 (30.8%)	20 (10.5%)	41 (16.7%)	39 (16.8%)	112 (15.8%)	
<1000 copies/mL	27 (69.2%)	171 (89.5%)	204 (83.3%)	193 (83.2%)	595 (84.2%)	
Low level viremia						.050
<200 c/mL	25 (64.1%)	163 (85.3%)	197 (80.4%)	181 (78.0%)	566 (80.1%)	
200–499 c/mL	0 (0.0%)	5 (2.6%)	4 (1.6%)	8 (3.4%)	17 (2.4%)	
500–999 c/mL	2 (5.1%)	3 (1.6%)	3 (1.2%)	4 (1.7%)	12 (1.7%)	
≥1000 c/mL	12 (30.8%)	20 (10.5%)	41 (16.7%)	39 (16.8%)	112 (15.8%)	

Data are presented as *n* (column %). Bold values indicate significance at *P* < 0.05. Pearson’s chi-squared tests were used to compare participants by age group. *P*-values were not corrected for multiple-hypothesis testing.

### Prevalence of Viral Suppression

The overall prevalence of VS was 0.84 (95% CI: 0.81–0.87; Table 3). The prevalence of VS was highest for 5–9-year-olds at 0.90 (95% CI: 0.84–0.93), followed by 10–14-year-olds at 0.83 (95% CI: 0.78–0.87) and 15–19-year-olds also at 0.83 (95% CI: 0.78–0.87) and then 1–4-year-olds at 0.69 (95% CI: 0.54–0.81). The prevalence of VS was highest among participants who had been on ART for 6–12 months at 0.89 (95% CI: 0.73–0.96), followed by those who had been on ART for 13–24 months at 0.86 (95% CI: 0.75–0.92) and then those who had been on ART for >24 months at 0.84 (95% CI: 0.81–0.86). The prevalence of VS among those on an INSTI-based regimen was 0.87 (95% CI: 0.84–0.90), compared to 0.77 (0.70–0.83) and 0.77 (0.64–0.86) for PI and NNRTI-based regimens respectively. Among the 595 with a VL < 1000 copies/mL, 566 (95.1%) had a VL < 200 copies/mL, 17 (2.9%) had a VL of 200–499 copies/mL and 12 (2.0%) had a VL of 500–999 copies/mL (Table 2).

**Table 3.** Prevalence of Viral Suppression among Children and Adolescents Attending MHRP/PEPFAR-Supported Antiretroviral Treatment Programs in Tanzania

	*N*	Prevalence of viral load < 1000 c/mL (95% CI)
Overall	595/707	0.84 (0.81–0.87)
Sex		
Male	271/325	0.83 (0.79–0.87)
Female	324/382	0.85 (0.81–0.88)
Age group		
1–4 years	27/39	0.69 (0.54–0.81)
5–9 years	171/191	0.90 (0.84–0.93)
10–14 years	204/245	0.83 (0.78–0.87)
15–19 years	193/232	0.83 (0.78–0.87)
Duration on ART		
6–12 months	25/28	0.89 (0.73–0.96)
13–24 months	54/63	0.86 (0.75–0.92)
>24 months	516/616	0.84 (0.81–0.86)
ART line		
1st line	342/405	0.84 (0.81–0.88)
2nd line	253/302	0.84 (0.79–0.88)
Current ART class		
NNRTI-based	43/56	0.77 (0.64–0.86)
PI-based	114/149	0.77 (0.70–0.83)
INSTI-based	438/502	0.87 (0.84–0.90)

Viral suppression was defined using WHO criteria as a viral load < 1000 copies/mL. The prevalence of viral suppression was estimated using the Wilson score method and reported with 95% confidence intervals (CIs) in the overall sample, and by sex, age group, and duration on ART, 1^st^ vs 2^nd^ line ART, and ART class.

### Factors Associated with Viral Suppression

Factors independently associated with VS included being on an INSTI-containing regimen as compared to an NNRTI or PI-containing regimen (aPR 1.15, 95% CI: 0.99–1.34; Table 4), age 5–9 years (aPR 1.16, 95% CI: 1.07–1.26) compared to other age groups, and seeking care at a referral health center (aPR 1.12, 95% CI: 1.04–1.21) as compared to a primary, district, or regional health facility. Factors inversely associated with VS included having one (aPR 0.82, 95% CI: 0.72–0.92) or two or more (aPR 0.79, 95% CI: 0.66–0.94) referrals for adherence counselling, and self-reporting missing one to two (aPR 0.88, 95% CI: 0.78–0.99) or three or more (aPR 0.77, 95% CI: 0.63–0.92) doses of ART in the past month as compared to missing no doses of ART.

**Table 4.** Factors Associated with Viral Suppression (Viral Load < 1000 copies/mL) among Children and Adolescents Attending MHRP/PEPFAR-Supported Antiretroviral Treatment Programs in Tanzania

	PR (95% CI)	aPR (95% CI)
Sex		
Male	Ref	Ref
Female	1.02 (0.95–1.08)	1.01 (0.95–1.07)
Age (years)		
1–4	0.83 (0.67–1.03)	0.96 (0.76–1.22)
5–9	1.08 (0.99–1.16)	**1.16 (1.07–1.26)**
10–14	Ref	Ref
15–19	1.00 (0.92–1.08)	1.02 (0.94–1.10)
Duration on ART		
≤24 months	Ref	Ref
>24 months	0.96 (0.88–1.05)	0.97 (0.90–1.05)
Current ART class		
NNRTI-based	Ref	Ref
PI-based	0.99 (0.84–1.18)	1.03 (0.87–1.21)
INSTI-based	1.14 (0.98–1.32)	1.15 (0.99–1.34)
Adherence counseling referrals		
Never referred	Ref	Ref
One referral	**0.82 (0.72–0.93)**	**0.82 (0.72–0.92)**
Two or more referrals	**0.82 (0.68–0.98)**	**0.79 (0.66–0.94)**
Self-reported frequency of taking medication		
Once per day	Ref	Ref
Twice or more per day	**0.93 (0.86–0.99)**	0.96 (0.87–1.06)
Self-reported missed doses of ART in past month		
None	Ref	Ref
1–2	**0.88 (0.78–0.99)**	**0.88 (0.78–0.99)**
3 or more	**0.77 (0.63–0.93)**	**0.77 (0.63–0.92)**
Level of care delivery		
Primary health facility	Ref	Ref
District health facility	0.97 (0.88–1.07)	0.97 (0.88–1.07)
Regional health facility	0.99 (0.88–1.12)	1.07 (0.96–1.20)
Referral health center	**1.11 (1.03–1.20)**	**1.12 (1.04–1.21)**

Bold values indicate significance at *P* < 0.05. *P*-values were not corrected for multiple-hypothesis testing. Generalized linear models with a Poisson distribution and robust standard errors were used to estimate unadjusted and adjusted prevalence ratios (aPRs) and 95% confidence intervals (CIs) for associations between sociodemographic and clinical factors and viral suppression. Individual level predictors determined to be significant (*α* = 0.05) in the unadjusted models along with predictors identified based on *a priori* and clinical knowledge of the study setting were included in the fully adjusted model. Predictors identified *a priori* included sex, age, duration on ART and current ART class. Additional predictors identified in bivariate analyses included adherence counseling referrals, frequency of taking medication, self-reported number of ART doses missed in the past month and facility level of care. We tested for multicollinearity using the variable inflation factor.

### HIV Drug Resistance Patterns

**Figure 1.**
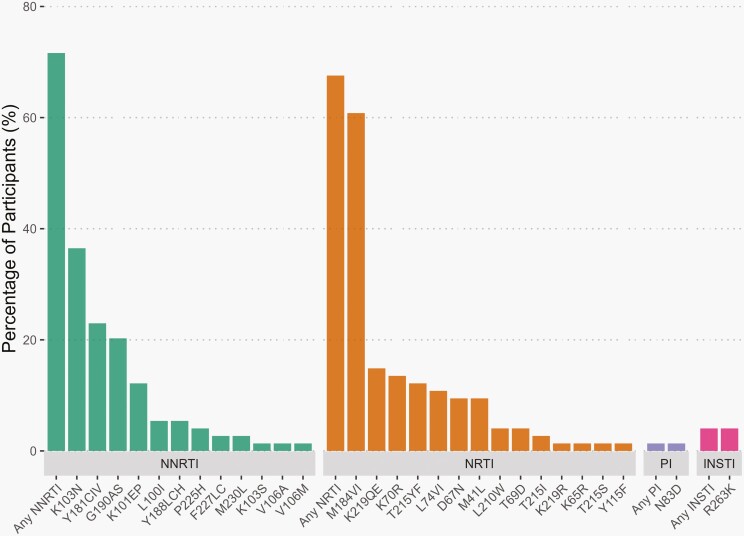
Frequency of NNRTI, NRTI, PI, and INSTI resistance mutations among participants with viral load ≥1000 copies/mL. Plasma samples from participants with viral load ≥1000 copies/mL underwent sequencing of the Pol region using a laboratory-validated modification to the ViroSeq HIV-1 Genotyping System v2.0 (Abbott Molecular, Chicago, IL). Sequences were evaluated for major mutations conferring resistance to nucleoside reverse transcriptase inhibitors (NRTIs), non-nucleoside reverse transcriptase inhibitors (NNRTIs), protease inhibitors (PIs), and integrase strand transfer inhibitors (INSTIs) using the SmartGene Integrated Database Network System (SmartGene, Zug, Switzerland) to access mutation lists from the Stanford HIV Drug Resistance Database Version 8.8.0 (Stanford University, Stanford, CA). The prevalence of specific drug resistance mutations and categories of drug resistance mutations were calculated by dividing the number of participants with one or more mutations by the total number of participants genotyped.

Of the 117 CALHIV who was unsuppressed (not restricted to complete cases), samples from 74 (63.3%) had successful PRRT and INT sequencing completed. Of these 74 participants, 60 (81.1%) had any major HIVDRM. In addition to their NRTI backbone, 12 (16.0%) were on an NNRTI; 21 (28.0%) on PI; and 41 (56.0%) on an INSTI-based ART regimen. Class-specific HIVDRM prevalence was 71.6% (*n* = 53) for NNRTIs, 67.6% (*n* = 50) for NRTIs, 1.4% (*n* = 1) for PIs and 4.1% (*n* = 3) for INSTIs. The K103N was the most common NNRTI mutation at 36.5% and the M184V/I was the most common NRTI mutation at 60.8%. The only PI-related mutation detected was the N83D and the only INSTI-related mutation detected was the R263K (Figure 1).

### HIV Genetic Diversity

**Figure 2.**
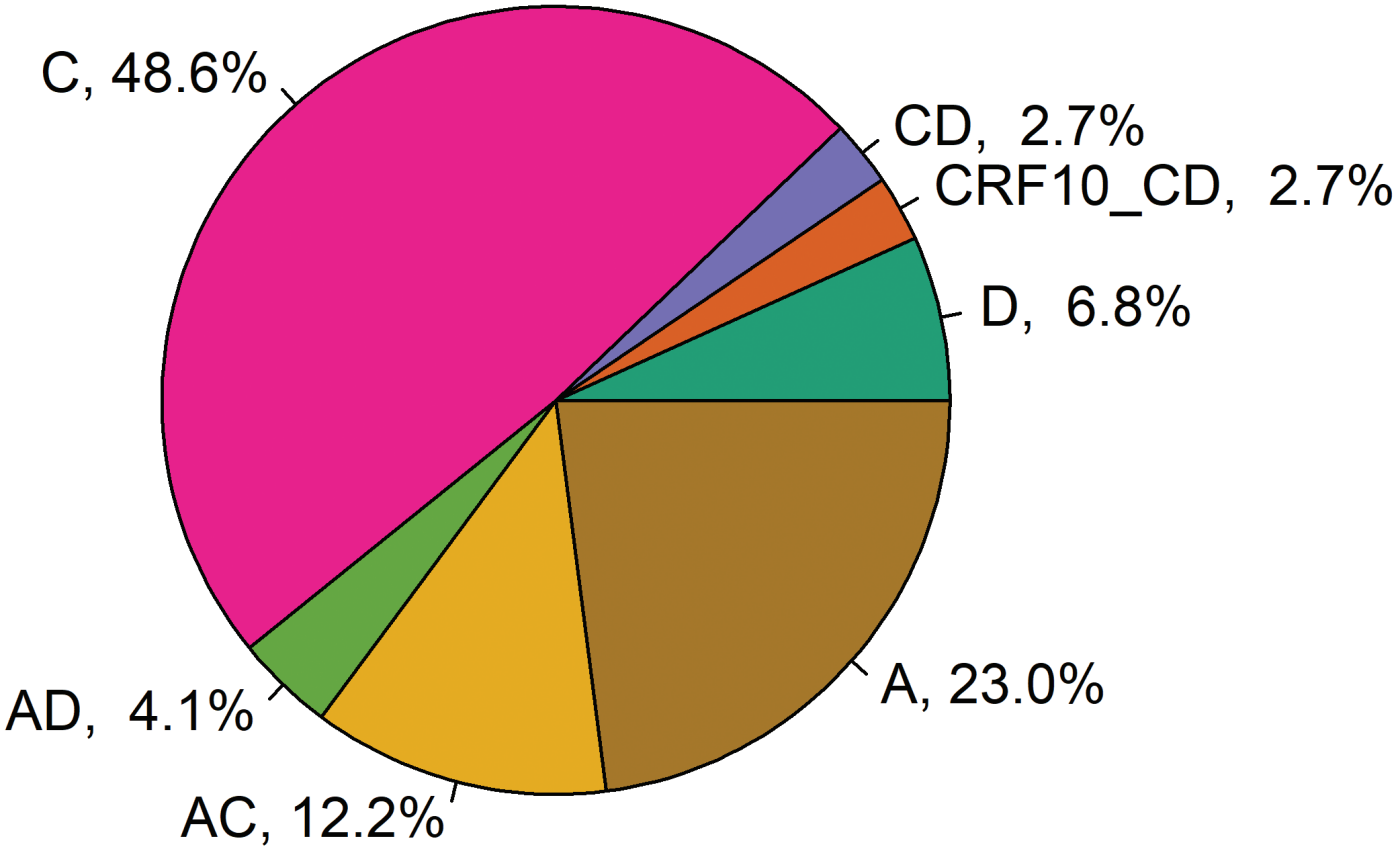
HIV-1 subtypes. HIV-1 subtype was inferred from the consensus evolutionary tree from SmartGene Integrated Database Network System, which utilizes the neighbor-joining method in MEGA4 software. The evolutionary distances were computed using the maximum composite likelihood method in units of the number of base substitutions per site. The tree was then generated by the neighbor-joining method from a nucleotide alignment.

The subtype distribution was heterogeneous with subtype C predominating (48.6%, *n* = 36 Figure 2). Other HIV-1 variants included A (23.0%, *n* = 11), D (6.8%, *n* = 5), and recombinant forms (17.3%, *n* = 13).

## DISCUSSION

This study is among the first to evaluate VS among CALHIV enrolled in routine care in public facilities in the era of DTG in Tanzania. We found 84% of participants to be virally suppressed. INSTI-containing regimens, age, and seeking/receiving care at referral health centers were independently associated with VS, while self-report of missed ART doses and referral for adherence counselling was associated with nonsuppression. Over 71%, 67%, and 4.1% of participants with viral non-suppression had major NNRTI, NRTI, and INSTI mutations respectively, with PI mutations remaining low at 1.3%.

Although still short of the desired 2020 UNAIDS goal of 90% and the 2030 goal of 95%, the overall 84% VS rate represents a significant improvement compared to earlier studies of children in Tanzania and East Africa. Recent studies of VS in African CALHIV have demonstrated much lower suppression rates (62–78%) [20–23]. A sister study conducted in Kenya that also sampled participants from MHRP-supported sites showed a VS rate of 80% [24]. This difference is likely due to the much higher utilization rates of DTG-containing regimens in the sites from this study compared to the Kenya study (71% vs 7%). It is unclear whether this is a programmatic difference or simply a reflection of this study being conducted approximately one year after the Kenyan study allowing for more time for pediatric ART regimen optimization. The data from this study supports clinical trial data that INSTI-containing regimens are superior to the previous standard of care [25].

Previous research has identified adolescents as having worse adherence and other key outcomes [26, 27]. This study showed that the pre-adolescent/early adolescent age range (10–14 years) was at higher risk of viral non-suppression compared to those between 5 and 9 years. This may be because of the recent transition of control in responsibility between the caregiver and the young teen, and the fact that they may decide to not take the medication in a fight for autonomy [28, 29] and also due to stigma and negative peer pressure, despite the implementation of adolescent-specific considerations interventions described in the Tanzanian treatment guidelines or increased utilization of INSTI-containing regimens. We also found CALHIV aged between 1 and 4 years to have the lowest VS rates (69%) among our cohort. In addition to medication use and adherence for this age group being fully dependent on caregivers, the non-availability of age-appropriate DTG formulations in Tanzania at the time of our study could be responsible for this finding. Although not significantly associated with VS in this study, we observed a decreasing trend for VS with increasing duration on ART. This finding may be connected to the development of treatment fatigue; a phenomenon that has been well characterized in literature among CALHIV [30–33] and frequently occurs with prolonged medication use in persons with chronic medical conditions.

We found that non-adherence negatively impacted VS in our study, with the youngest age band reporting the highest percentages of missed doses of ART in the month prior to enrollment. Effective long-term solutions for medication adherence remain elusive among CALHIV and continue to be a priority for treatment programs. The Tanzania guidelines recommend adherence assessments at every clinic visit. These sessions explore reasons for poor adherence, the patient’s support system, and medication routine, with modifications to meet client-based needs at the facility and community levels [12]. Despite these guidelines, this study’s results, as well as the sister study in Kenya, which has a similar enhanced adherence counseling (EAC) program, showed relatively high numbers of referrals for adherence counseling and self-reported missed doses of ART. There are several possible explanations for this. The current EAC guidelines may need further revision to better understand and address barriers to adherence [34]. There are many socioeconomic and structural factors that impact adherence and are outside the scope of the EAC sessions. Another possibility is that current adherence guidelines are not being met due to resource constraints. This was potentially demonstrated in this study and the sister Kenya study that showed, along with an increase in adherence referrals, smaller facilities with fewer resources were also associated with lower rates of VS [24].

Adherence to ART for CALHIV may remain suboptimal until more pediatric-friendly treatment regimens become globally available. Palatability and complex dosing regimens have consistently been shown to greatly affect pediatric adherence [35]. Recent clinical trials investigating fixed-dose ART combinations in children may provide some future relief for this issue [24]. Nonetheless, adherence may not be the only driving factor associated with failed VS. Data from recent studies have started to define lower thresholds than the previously accepted 95% adherence needed for 90% VS. In 2019, Byrd et al. estimated that an 82% adherence level would be needed to achieve similar VS levels [36]. Due to the fact that children under 30 kg require different dosing as they grow, it is possible that some children are inadequately dosed for some period of time, which may contribute to failed VS despite optimal adherence.

We found a widespread occurrence of NNRTI and NRTI mutations among our participants with viral non-suppression. Prior to the rollout of DTG-based regimens, efavirenz and nevirapine-based regimens were the mainstay of ART in resource-limited settings. The migration to more optimal drugs was informed by the low-genetic barrier of and early emergence of HIVDRMs to NNRTIs. Despite the positive association between INSTI-regimens and VS reported in this study, we found a 4.1% prevalence of the INSTI-related R263K; a nonpolymorphic mutation selected *in vitro* by DTG and other INSTIs [37]. A recent study using a nationally representative sample of adolescents and young adults from Tanzania reported a prevalence of 6.3% for INSTI HIVDRMs [38]. Taken together, these findings indicate the need for optimizing patient-level outcomes and continued surveillance for INSTI-specific HIVDRMs within the context of optimized ART regimens in resource-limited settings. Studies have shown that HIV variants definitively or potentially conferring INSTI resistance rarely occur in patients that are INSTI naïve. However longitudinal resistance studies to track the potential emergence of INSTI-HIVDRMs are needed as these drugs continue to be rolled out as the preferred first-line regimens [23, 24].

### Limitations

Due to the cross-sectional study design, only associations with regard to VS could be made. Follow-up studies to assess trends in VS are needed. Since this study was conducted only in PEPFAR-supported sites, it may lack generalizability to other areas with different levels of resources. Additionally, this study’s method for measuring adherence by self or caregiver report likely underestimates the true rate of nonadherence [39]. Specific resistance testing for DTG and INSTIs was unavailable for this study, preventing any conclusions about INSTI resistance in our cohort. HIVDRM testing among those on INSTI regimens with nonsuppression will provide valuable information.

## CONCLUSIONS

This study demonstrated 84% VS prevalence among CALHIV in Tanzania; a significant improvement compared to earlier studies in East Africa. Further exploration of associations with failed VS shown in the study may suggest potential interventions to bring the VS rate up to the UNAIDS’ goal of ≥95% VS. Prospective studies focused on areas such as the effectiveness of the EAC program, characteristics of those failing on INSTI-regimens and the quality of care at lower level facilities would potentially allow for interventions that provide better, patient-centered care for those still not meeting VS thresholds. Regardless, this study supports WHO guidelines on the need to optimize children on INSTI-containing regimens.

## Supplementary Material

piad040_suppl_Supplementary_Figure_S1

piad040_suppl_Supplementary_Table_S1

piad040_suppl_Supplementary_Figure_Legend

## List and definition of all abbreviations (in alphabetical order):

3TC: Lamivudine
ABC: Abacavir
AIDS: Acquired immunodeficiency syndrome
α: Alpha
aPR: Adjusted prevalence ratio
ART: Antiretroviral therapy
ATV/r: Atazanavir boosted with ritonavir
AZ: Arizona
AZT: Zidovudine
CA: California
CALHIV: Children and adolescents living with HIV
CIs: Confidence intervals
DNA: Deoxyribonucleic acid
DTG: Dolutegravir
EAC: Enhanced adherence counseling
HIV: Human immunodeficiency virus
HIVDRMs: HIV drug resistance mutations
HJFMRI: Henry Jackson Foundation Medical Research International
IDNS: Integrated database network system
IL: Illinois
INT: Integrase
INSTI: Integrase strand transfer inhibitor
IQR: Interquartile range
Kg: Kilogram
LPV/r: Lopinavir boosted with ritonavir
MHRP: Military HIV Research Program
mL: Milliliter
NCBI: National center for biotechnology information
NNRTI: Non-nucleoside reverse transcriptase inhibitor
NRTIs: Nucleoside reverse transcriptase inhibitors
PCR: Polymerase chain reaction
PEPFAR: US President’s Emergency Plan for AIDS Relief
PIs: Protease inhibitors
PR: Protease
QC: Quality control
REGA: HIV subtyping tool of the same as acronym
RNA: Ribonucleic acid
RT: Reverse transcriptase
RV517: Retrovirology 517
SAS: Statistical Analysis System
STATA: Statistical software known by its acronym
TB: Tuberculosis
TDF: Tenofovir disoproxil fumarate
UNAIDS: Joint United Nations Program on HIV/AIDS
U.S: United States
USA: United States of America
VL: Viral load
VS: Viral suppression
WHO: World Health Organization
AZT: Zidovudine

## Notes

***Financial support.*** This work was supported by U.S. President’s Emergency Plan for AIDS Relief/Office of the Global AIDS Coordinator via a cooperative agreement between the Henry M. Jackson Foundation for the Advancement of Military Medicine, Inc., and the U.S. Department of Defense (grant number W81XWH-11-2-0174). This publication was also supported by the National Institute of Allergy and Infectious Diseases of the National Institutes of Health (award # T32AI114398).

***Potential conflicts of interest.*** The authors have no potential conflicts of interest to disclose.

***Disclaimer.*** The views expressed are those of the authors and do represent the positions of the U.S. Army, the Department of Defense, the Uniformed Services University, the Henry M. Jackson Foundation for the Advancement of Military Medicine, or the National Institutes of Health. The investigators have adhered to the policies for the protection of human subjects as prescribed in Army Regulation 70-25.
