# Supplementary figures and images for: Factors Associated With Viral Suppression and Drug Resistance in Children and Adolescents Living With HIV in Care and Treatment Programs in Southern Tanzania
Source: J Pediatric Infect Dis Soc. 2023 Jun 3;12(6):353–63. doi: 10.1093/jpids/piad040 (PMC10312299; doi:10.1093/jpids/piad040)

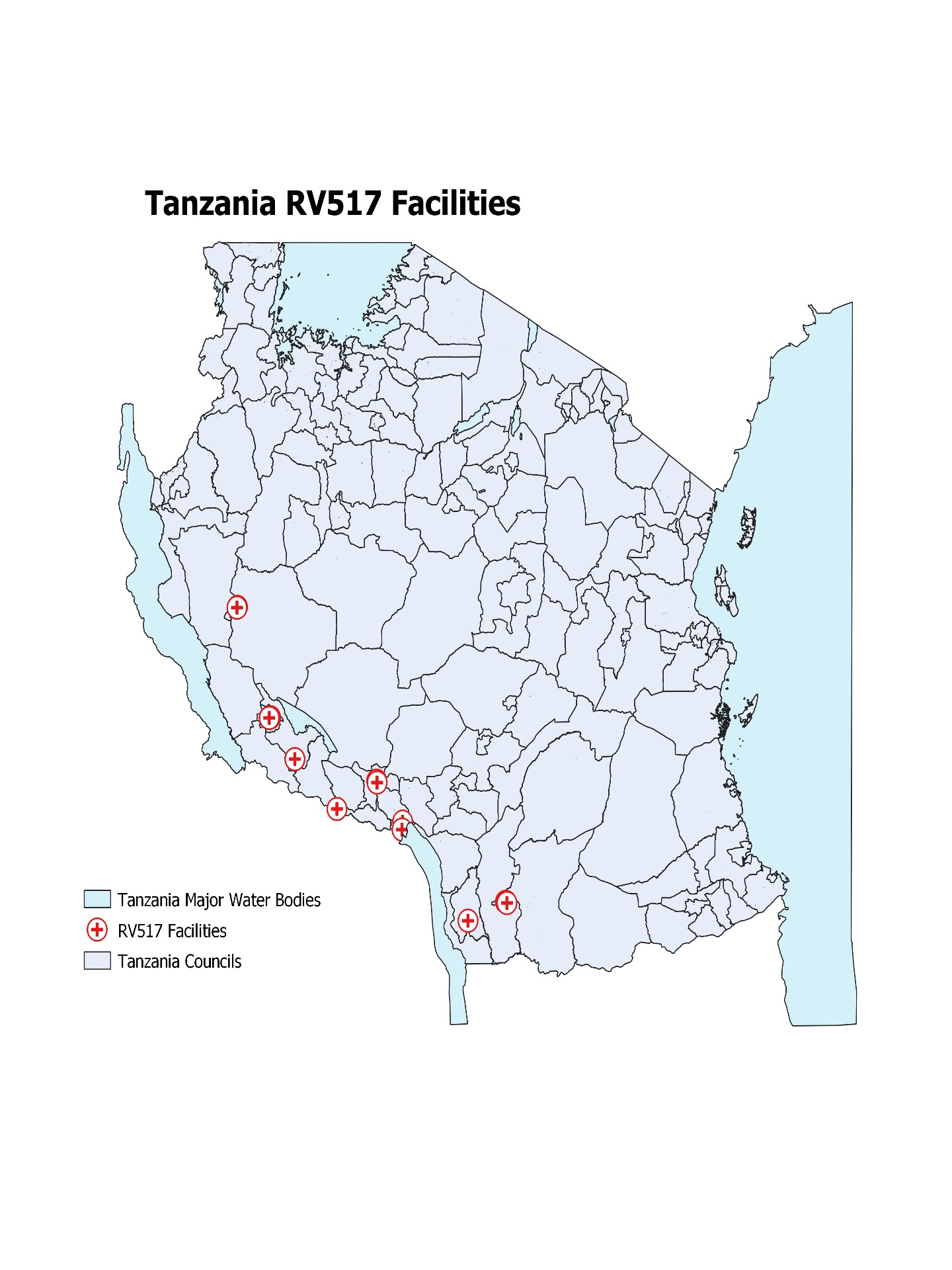

Supplement: piad040_suppl_Supplementary_Figure_S1 [file piad040_suppl_supplementary_figure_s1.jpeg]
