# Supplementary material for: Factors Associated With Viral Suppression and Drug Resistance in Children and Adolescents Living With HIV in Care and Treatment Programs in Southern Tanzania
Source: J Pediatric Infect Dis Soc. 2023 Jun 3;12(6):353–63. doi: 10.1093/jpids/piad040 (PMC10312299; doi:10.1093/jpids/piad040)
Supplement: piad040_suppl_Supplementary_Table_S1 [file piad040_suppl_supplementary_table_s1.docx]

Supplemental Table. ART Regimen by Weight Band

|  | Weight Band | | |  |
| --- | --- | --- | --- | --- |
|  | <20 kg | 20-20.9 kg | 30+ kg | Total |
|  | (n=106) | (n=202) | (n=397) | N=705* |
| Current ART regimen |  |  |  |  |
| ABC/3TC/EFV | 5 (4.7%) | 8 (4.0%) | 3 (0.8%) | 16 (2.3%) |
| AZT/3TC/EFV | 0 (0.0%) | 8 (4.0%) | 2 (0.5%) | 10 (1.4%) |
| ABC/3TC/LPV/r | 78 (73.6%) | 22 (10.9%) | 7 (1.8%) | 107 (15.2%) |
| AZT/3TC/LPV/r | 1 (0.9%) | 1 (0.5%) | 1 (0.3%) | 3 (0.4%) |
| TDF/3TC/ATV/r | 0 (0.0%) | 0 (0.0%) | 16 (4.0%) | 16 (2.3%) |
| TDF/3TC/DTG | 2 (1.9%) | 13 (6.4%) | 324 (81.6%) | 339 (48.1%) |
| AZT/3TC/NVP | 10 (9.4%) | 11 (5.4%) | 7 (1.8%) | 28 (4.0%) |
| AZT/3TC/ATV/r | 1 (0.9%) | 2 (1.0%) | 1 (0.3%) | 4 (0.6%) |
| TDF/3TC/EFV | 0 (0.0%) | 1 (0.5%) | 0 (0.0%) | 1 (0.1%) |
| ABC/3TC/ATV/r | 0 (0.0%) | 0 (0.0%) | 13 (3.3%) | 13 (1.8%) |
| ABC/3TC/DTG | 8 (7.5%) | 133 (65.8%) | 12 (3.0%) | 153 (21.7%) |
| AZT/3TC/DTG | 1 (0.9%) | 3 (1.5%) | 5 (1.3%) | 9 (1.3%) |
| TDF/3TC/LPV/r | 0 (0.0%) | 0 (0.0%) | 5 (1.3%) | 5 (0.7%) |
| TDF/3TC/NVP | 0 (0.0%) | 0 (0.0%) | 1 (0.3%) | 1 (0.1%) |

Abbreviations: ABC, abacavir; AZT, azidothymidine (zidovudine); 3TC, lamivudine; EFV, efavirenz; DTG, dolutegravir; NVP, nevirapine; TDF, tenofovir; ATV/r, atazanavir/ritonavir; LPV/r, lopinavir/ritonavir.

*Note: n=2 missing weight data
